# Supplementary material for: Improving timeliness in the neglected tropical diseases preventive chemotherapy donation supply chain through information sharing: A retrospective empirical analysis
Source: PLoS Negl Trop Dis. 2021 Nov 29;15(11):e0009523. doi: 10.1371/journal.pntd.0009523 (PMC8659369; doi:10.1371/journal.pntd.0009523)
Supplement: S1 Table — (DOCX) [file pntd.0009523.s001.docx]

**S1 Table. Dummy coding matrices**

Dummy coding for medicine control variable

| Categories of original variable | Dummy variable coding  *X*_1_*X*_2_*X*_3_ | | | Description of dummy variable |
| --- | --- | --- | --- | --- |
|  | **M1** | **M2** | **M3** |  |
| Med2 | 1 | 0 | 0 | 1 = Med2, 0 = otherwise |
| Med3 | 0 | 1 | 0 | 1 = Med3, 0 = otherwise |
| Med4 | 0 | 0 | 1 | 1 = Med4, 0 = otherwise |
| Med1 | 0 | 0 | 0 | All 0’s = Med 1 |

Dummy coding for region control variable

| Categories of Original Variable | Dummy Variable Coding  *X*_1_*X*_2_*X*_3_ *X*_4_ *X*_5_ | | | | | Description of Dummy Variable |
| --- | --- | --- | --- | --- | --- | --- |
|  | **R1** | **R2** | **R3** | **R4** | **R5** |  |
| AMRO | 1 | 0 | 0 | 0 | 0 | 1 = Region1, 0 = otherwise |
| EMRO | 0 | 1 | 0 | 0 | 0 | 1 = Region2, 0 = otherwise |
| EURO | 0 | 0 | 1 | 0 | 0 | 1 = Region3, 0 = otherwise |
| SEARO | 0 | 0 | 0 | 1 | 0 | 1 = Region4, 0 = otherwise |
| WPRO | 0 | 0 | 0 | 0 | 1 | 1 = Region5, 0 = otherwise |
| AFRO | 0 | 0 | 0 | 0 | 0 |  |

Dummy coding for disease control variable

| Categories of Original Variable | Dummy Variable Coding  *X*_1_*X*_2_ | | Description of Dummy Variable |
| --- | --- | --- | --- |
|  | **D1** | **D2** |  |
| Disease1 | 1 | 0 | 1 = Disease1, 0 = otherwise |
| Disease2 | 0 | 1 | 1 = Disease2, 0 = otherwise |
| Disease0 | 0 | 0 |  |

Dummy coding for order size control variable

| Categories of Original Variable | Dummy Variable Coding  *X*_1_*X*_2_*X*_3_ | | Description of Dummy Variable |
| --- | --- | --- | --- |
|  | **T1** | **T2** |  |
| 10m > X ≥ 3m | 1 | 0 | 1 = order size 10m > X ≥ 3m  0 = otherwise |
| X < 3m | 0 | 1 | 1 = order size X <3m  0 = otherwise |
| X ≥ 10m | 0 | 0 |  |

Dummy coding for shipment mode control variable

| Categories of Original Variable | Dummy Variable Coding  *X*_1_*X*_2_ | | Description of Dummy Variable |
| --- | --- | --- | --- |
|  | **S1** | **S2** |  |
| Sea | 1 | 0 | 1 = sea, 0 = otherwise |
| Land | 0 | 1 | 1 = land, 0 = otherwise |
| Air | 0 | 0 |  |
