# Supplementary material for: Improving timeliness in the neglected tropical diseases preventive chemotherapy donation supply chain through information sharing: A retrospective empirical analysis
Source: PLoS Negl Trop Dis. 2021 Nov 29;15(11):e0009523. doi: 10.1371/journal.pntd.0009523 (PMC8659369; doi:10.1371/journal.pntd.0009523)
Supplement: S2 Table — (DOCX) [file pntd.0009523.s002.docx]

**S2 Table. International Logistics Performance Index (LPI) Control Variable Assignment Details**

To incorporate the LPI, the overall LPI score for a given country was included in the data. The LPI is not assessed every year, therefore the score for the year of assessment closest to the year of the purchase order was included and assigned according to the table below.

| Purchase Order Year | LPI Year |
| --- | --- |
| 2006 | 2007 |
| 2007 | 2007 |
| 2008 | 2007 |
| 2009 | 2010 |
| 2010 | 2010 |
| 2011 | 2012 |
| 2012 | 2012 |
| 2013 | 2014 |
| 2014 | 2014 |
| 2015 | 2016 |
| 2016 | 2016 |
| 2017 | 2018 |
| 2018 | 2018 |
