# Supplementary material for: Improving timeliness in the neglected tropical diseases preventive chemotherapy donation supply chain through information sharing: A retrospective empirical analysis
Source: PLoS Negl Trop Dis. 2021 Nov 29;15(11):e0009523. doi: 10.1371/journal.pntd.0009523 (PMC8659369; doi:10.1371/journal.pntd.0009523)
Supplement: S3 Table — (DOCX) [file pntd.0009523.s003.docx]

**S3 Table. Full linear regression results**

Linear regression results – PO timeliness

| **Model** | | **Unstandardized Coefficients** | | **Standardized Coefficients** | **t** | **Sig.** | **95.0% Confidence Interval for B** | |
| --- | --- | --- | --- | --- | --- | --- | --- | --- |
|  |  | **B** | **Std. Error** | **Beta** |  |  | **Lower Bound** | **Upper Bound** |
|  | (Constant) | 2.064 | 1.352 |  | 1.527 | .127 | -.590 | 4.718 |
|  | Pre vs Post SCPMS | .941 | .313 | .098 | 3.006 | .003 | .327 | 1.555 |
|  | LPI Indicator | -1.645 | .495 | -.142 | -3.323 | <.001 | -2.617 | -.674 |
|  | R1 - Region Dummy | 2.387 | .625 | .133 | 3.816 | <.001 | 1.159 | 3.614 |
|  | R2 - Region Dummy | 1.030 | .707 | .049 | 1.457 | .146 | -.358 | 2.417 |
|  | R3 - Region Dummy | 1.227 | 1.306 | .032 | .940 | .348 | -1.336 | 3.790 |
|  | R4 - Region Dummy | 1.303 | .507 | .126 | 2.571 | .010 | .309 | 2.298 |
|  | R5 - Region Dummy | 3.231 | .565 | .207 | 5.721 | <.001 | 2.122 | 4.339 |
|  | M1 - Medicine Dummy | .901 | .511 | .064 | 1.764 | .078 | -.101 | 1.903 |
|  | M2 - Medicine Dummy | 1.345 | .567 | .090 | 2.373 | .018 | .233 | 2.457 |
|  | D1 - Disease Dummy | 1.510 | .491 | .118 | 3.073 | .002 | .546 | 2.475 |
|  | D2 - Disease Dummy | -.432 | .427 | -.044 | -1.010 | .313 | -1.270 | .407 |
|  | T1 - Tablets Dummy | .089 | .427 | .009 | .209 | .835 | -.749 | .927 |
|  | T2 - Tablets Dummy | -.126 | .582 | -.011 | -.217 | .828 | -1.268 | 1.015 |
|  | S1 - Shipment Dummy | 1.374 | .468 | .148 | 2.933 | .003 | .455 | 2.294 |
|  | S2 - Shipment Dummy | .649 | .730 | .051 | .889 | .374 | -.784 | 2.083 |

Linear regression results – go signal timeliness

| **Model** | | **Unstandardized Coefficients** | | **Standardized Coefficients** | **t** | **Sig.** | **95.0% Confidence Interval for B** | |
| --- | --- | --- | --- | --- | --- | --- | --- | --- |
|  |  | B | Std. Error | Beta |  |  | Lower Bound | Upper Bound |
|  | (Constant) | -130.324 | 42.718 |  | -3.051 | .003 | -214.557 | -46.092 |
|  | Pre vs Post SCPMS | 41.483 | 10.673 | .281 | 3.887 | <.001 | 20.438 | 62.529 |
|  | LPI Indicator | 33.066 | 14.313 | .154 | 2.310 | .022 | 4.843 | 61.289 |
|  | R1 - Region Dummy | 39.379 | 15.564 | .197 | 2.530 | .012 | 8.689 | 70.069 |
|  | R2 - Region Dummy | 17.335 | 17.285 | .072 | 1.003 | .317 | -16.749 | 51.419 |
|  | R3 - Region Dummy | 30.035 | 22.593 | .097 | 1.329 | .185 | -14.516 | 74.585 |
|  | R4 - Region Dummy | 17.256 | 18.172 | .071 | .950 | .343 | -18.577 | 53.088 |
|  | R5 - Region Dummy | 14.357 | 18.372 | .056 | .781 | .435 | -21.869 | 50.583 |
|  | M1 - Medicine Dummy | 45.060 | 71.897 | .041 | .627 | .532 | -96.710 | 186.830 |
|  | M2 - Medicine Dummy | 35.545 | 15.297 | .209 | 2.324 | .021 | 5.381 | 65.709 |
|  | D1 - Disease Dummy | 14.813 | 16.161 | .079 | .917 | .360 | -17.054 | 46.679 |
|  | D2 - Disease Dummy | -36.620 | 14.842 | -.246 | -2.467 | .014 | -65.885 | -7.355 |
|  | T1 - Tablets Dummy | 14.953 | 16.866 | .101 | .887 | .376 | -18.304 | 48.210 |
|  | T2 - Tablets Dummy | 26.709 | 19.943 | .170 | 1.339 | .182 | -12.615 | 66.033 |
|  | S1 - Shipment Dummy | 26.598 | 14.586 | .178 | 1.824 | .070 | -2.163 | 55.358 |

Linear regression results – shipment timeliness

| **Model** | | **Unstandardized Coefficients** | | **Standardized Coefficients** | **t** | **Sig.** | **95.0% Confidence Interval for B** | |
| --- | --- | --- | --- | --- | --- | --- | --- | --- |
|  |  | **B** | **Std. Error** | **Beta** |  |  | **Lower Bound** | **Upper Bound** |
|  | (Constant) | -.908 | 1.578 |  | -.575 | .565 | -4.009 | 2.193 |
|  | Pre vs Post SCPMS | .033 | .360 | .004 | .091 | .927 | -.675 | .740 |
|  | R1 - Region Dummy | -.788 | .564 | -.074 | -1.397 | .163 | -1.895 | .320 |
|  | R2 - Region Dummy | 2.425 | .703 | .151 | 3.452 | <.001 | 1.045 | 3.805 |
|  | R3 - Region Dummy | -.027 | .752 | -.002 | -.035 | .972 | -1.505 | 1.452 |
|  | R4 - Region Dummy | .086 | 1.259 | .003 | .068 | .946 | -2.388 | 2.560 |
|  | R5 - Region Dummy | .122 | .573 | .012 | .213 | .832 | -1.003 | 1.247 |
|  | M1 - Medicine Dummy | 3.329 | .641 | .230 | 5.189 | <.001 | 2.069 | 4.589 |
|  | M2 - Medicine Dummy | 4.191 | 1.045 | .171 | 4.011 | <.001 | 2.139 | 6.244 |
|  | D1 - Disease Dummy | 2.860 | .547 | .257 | 5.229 | <.001 | 1.786 | 3.934 |
|  | D2 - Disease Dummy | -.097 | .598 | -.008 | -.162 | .871 | -1.273 | 1.079 |
|  | T1 - Tablets Dummy | -.443 | .451 | -.053 | -.981 | .327 | -1.329 | .444 |
|  | T2 - Tablets Dummy | .267 | .480 | .032 | .556 | .578 | -.675 | 1.209 |
|  | S1 - Shipment Dummy | -.168 | .704 | -.016 | -.239 | .811 | -1.552 | 1.215 |
|  | S2 - Shipment Dummy | .381 | .528 | .045 | .722 | .471 | -.656 | 1.418 |

Linear regression results – arrival timeliness

| **Model** | | **Unstandardized Coefficients** | | **Standardized Coefficients** | **t** | **Sig.** | **95.0% Confidence Interval for B** | |
| --- | --- | --- | --- | --- | --- | --- | --- | --- |
|  |  | **B** | **Std. Error** | **Beta** |  |  | **Lower Bound** | **Upper Bound** |
|  | (Constant) | 1.941 | 1.429 |  | 1.358 | .175 | -.866 | 4.747 |
|  | Pre vs Post SCPMS | .516 | .339 | .058 | 1.522 | .129 | -.150 | 1.182 |
|  | R1 - Region Dummy | -1.577 | .513 | -.144 | -3.073 | .002 | -2.586 | -.569 |
|  | R2 - Region Dummy | 1.679 | .667 | .101 | 2.517 | .012 | .369 | 2.990 |
|  | R3 - Region Dummy | -.452 | .776 | -.023 | -.583 | .560 | -1.976 | 1.071 |
|  | R4 - Region Dummy | .270 | 1.287 | .008 | .210 | .834 | -2.256 | 2.797 |
|  | R5 - Region Dummy | .748 | .529 | .075 | 1.413 | .158 | -.291 | 1.787 |
|  | M1 - Medicine Dummy | 2.965 | .566 | .220 | 5.238 | <.001 | 1.853 | 4.077 |
|  | M2 - Medicine Dummy | 1.250 | .565 | .092 | 2.213 | .027 | .141 | 2.358 |
|  | D1 - Disease Dummy | 2.648 | .555 | .210 | 4.771 | <.001 | 1.558 | 3.738 |
|  | D2 - Disease Dummy | -.276 | .515 | -.024 | -.537 | .592 | -1.287 | .734 |
|  | T1 - Tablets Dummy | -.365 | .446 | -.042 | -.819 | .413 | -1.241 | .510 |
|  | T2 - Tablets Dummy | -.096 | .457 | -.011 | -.210 | .834 | -.993 | .801 |
|  | S1 - Shipment Dummy | -.854 | .667 | -.084 | -1.281 | .200 | -2.163 | .455 |
|  | S2 - Shipment Dummy | -.310 | .499 | -.037 | -.622 | .534 | -1.289 | .669 |

Linear regression results – delivery timeliness

| **Model** | | **Unstandardized Coefficients** | | **Standardized Coefficients** | **t** | **Sig.** | **95.0% Confidence Interval for B** | |
| --- | --- | --- | --- | --- | --- | --- | --- | --- |
|  |  | **B** | **Std. Error** | **Beta** |  |  | **Lower Bound** | **Upper Bound** |
|  | (Constant) | .112 | 1.670 |  | .067 | .947 | -3.169 | 3.393 |
|  | Pre vs Post SCPMS | .828 | .373 | .093 | 2.220 | .027 | .095 | 1.560 |
|  | R1 - Region Dummy | -1.000 | .591 | -.094 | -1.694 | .091 | -2.161 | .160 |
|  | R2 - Region Dummy | 1.752 | .783 | .100 | 2.239 | .026 | .215 | 3.290 |
|  | R3 - Region Dummy | -.320 | .790 | -.018 | -.405 | .686 | -1.873 | 1.232 |
|  | R4 - Region Dummy | 2.664 | 1.633 | .070 | 1.631 | .103 | -.545 | 5.873 |
|  | R5 - Region Dummy | .062 | .634 | .006 | .098 | .922 | -1.184 | 1.308 |
|  | M1 - Medicine Dummy | 2.945 | .677 | .199 | 4.352 | <.001 | 1.615 | 4.274 |
|  | M2 - Medicine Dummy | 4.596 | 1.258 | .157 | 3.654 | <.001 | 2.125 | 7.067 |
|  | D1 - Disease Dummy | 2.469 | .595 | .209 | 4.151 | <.001 | 1.301 | 3.638 |
|  | D2 - Disease Dummy | .002 | .561 | .000 | .003 | .998 | -1.100 | 1.103 |
|  | T1 - Tablets Dummy | .350 | .498 | .040 | .704 | .482 | -.628 | 1.328 |
|  | T2 - Tablets Dummy | .087 | .506 | .010 | .171 | .864 | -.907 | 1.080 |
|  | S1 - Shipment Dummy | -.331 | .742 | -.031 | -.446 | .656 | -1.788 | 1.126 |
|  | S2 - Shipment Dummy | -.507 | .557 | -.058 | -.911 | .363 | -1.601 | .586 |
