# Supplementary material for: Improving timeliness in the neglected tropical diseases preventive chemotherapy donation supply chain through information sharing: A retrospective empirical analysis
Source: PLoS Negl Trop Dis. 2021 Nov 29;15(11):e0009523. doi: 10.1371/journal.pntd.0009523 (PMC8659369; doi:10.1371/journal.pntd.0009523)
Supplement: S4 Table — (DOCX) [file pntd.0009523.s004.docx]

**S4 Table. Robustness check results**

Summary of ordinary least squares regression results applying a six-month lag in the information sharing effect

| Dependent Variable | PO timeliness  (in months) | Go signal timeliness  (in days) | Shipment timeliness  (in months) | Arrival timeliness  (in months) | Delivery timeliness  (in months) |
| --- | --- | --- | --- | --- | --- |
| NTDeliver | 0.999 | 38.495 | - 0.026 | 0.204 | 0.741 |
| p-value | 0.002 | 0.000 | 0.937 | 0.553 | 0.046 |
| WHO region | Yes | Yes | Yes | Yes | Yes |
| Medicine type | Yes | Yes | Yes | Yes | Yes |
| Disease | Yes | Yes | Yes | Yes | Yes |
| Order size | Yes | Yes | Yes | Yes | Yes |
| Mode of shipment | Yes | No* | Yes | Yes | Yes |
| Observations | 970 | 226 | 569 | 715 | 567 |
| *Sample size too limited to incorporate variable | | | | | |

Summary of ordinary least squares regression results applying a “double pretest”

| Dependent Variable | PO timeliness  (in months) | Go signal timeliness  (in days) | Shipment timeliness  (in months) | Arrival timeliness  (in months) | Delivery timeliness  (in months) |
| --- | --- | --- | --- | --- | --- |
| NTDeliver | 1.42 | -30.476 | 0.991 | 0.581 | 0.173 |
| p-value | 0.000 | 0.293 | 0.215 | 0.203 | 0.818 |
| WHO region | Yes | Yes | Yes | Yes | Yes |
| Medicine type | Yes | Yes | Yes | Yes | Yes |
| Disease | Yes | Yes | Yes | Yes | Yes |
| Order size | Yes | Yes | Yes | Yes | Yes |
| Mode of shipment | Yes | No* | Yes | Yes | Yes |
| Observations | 664 | 117 | 339 | 520 | 380 |
| *Sample size too limited to incorporate variable | | | | | |
