# Supplementary material for: Improving timeliness in the neglected tropical diseases preventive chemotherapy donation supply chain through information sharing: A retrospective empirical analysis
Source: PLoS Negl Trop Dis. 2021 Nov 29;15(11):e0009523. doi: 10.1371/journal.pntd.0009523 (PMC8659369; doi:10.1371/journal.pntd.0009523)
Supplement: Attachment — Submitted filename: NTD PLOS Response Letter to Reviewers_Elena_Kasparis_FINAL.docx [file pntd.0009523.s005.docx]

**PNTD-D-21-00785:**

**“Improving Timeliness in the Neglected Tropical Diseases Preventive Chemotherapy Donation Supply Chain through Information Sharing: A Retrospective Empirical Analysis”**

**I: General Response to Editor and Reviewers**

We wish to sincerely thank the Associate Editor Gregory Deye, the Deputy Editor Bruce Lee, and the reviewers for the opportunity to revise our paper and are grateful for the thoughtful reviews and constructive comments, which helped us improve our paper substantially.

In the current submission, we have carefully revised the paper focusing on (i) improving the explanation of our research method and design, ii) clarifying the analysis and making some additions as requested, and iii) providing more clarity on the importance of the research and its contribution to the field of supply chain management in global health. We have also addressed various other concerns and minor edits that were raised by the reviewing team. We next provide detailed responses to feedback comments.

**II: Detailed Response to Reviewer 1**

***“Overall, this is an interesting manuscript that attempts to utilize a pre-post design to evaluate the potential impact of the NTDeliver platform on improving supply chain management for PC diseases. The findings are of interest to the NTD funder, policy and program community and fit well within the scope of this journal. There are substantial issues with the way in which the paper is presented and written that need to be addressed. In addition, the authors need to tone down the language implying causality, as this study design can only assess associations, not causality. If these changes are made, I believe the paper will be a useful contribution to the literature.”***

Thank you for the complimentary feedback regarding the potential value of this manuscript to the intended audience of the various NTD program stakeholders relevant to *PLOS Neglected Tropical Diseases*. Updates to the paper presentation and writing have been made in accordance with the specific feedback provided below and these changes have been specified in the response to each feedback note.

In terms of the note on revising the language implying causality, the following changes made to the manuscript to reflect this feedback:

- Revisions to the abstract and introduction, such as changing the wording from *“information sharing has a positive impact”* to *“information sharing has a positive association”*
- Revisions in the results section were made, such as changing wording from “significant impact” to “significant association”
- Revisions to the discussion were made to soften any insinuation of confirmed causality, more accurately reflect the validity of the results, and the contribution of this paper, such as *“our research results certainly helps to validate the positive and measurable impact of the information intervention on supply chain performance”* to *“our research results certainly helps to validate the positive association between information sharing and supply chain performance”*

***“A critical limitation of this analysis is that it only evaluates the impact on the “first mile”. It is well known that many of the delays in getting MDA drugs to communities happen between the central store and the program. As such, it is difficult to know how much of an impact this platform actually has on drug delivery.”***

Thank you for this valuable comment. While the last mile certainly involves many challenges that contribute to MDA delays, issues with the first mile have also shown in practice to have a downstream effect on the last mile and ultimately MDA timeliness. Research exists reviewing this impact of first mile delays on MDA timeliness, specifically, the outcomes of Koporc et al (2015)’s research referenced in our manuscript [1]. From Koporc et al’s (2015) research, it is suggested that primary causes for delayed and missed MDAs may occur both from in-country issues as well as upstream production challenges, with customs issues contributing. Therefore, Koporc et al.’s research supports a potential relationship between first mile supply chain issues and timeliness of MDAs. As another consideration, last mile issues are also very country, context, and disease or health program-specific. It is not the intent of this first-mile analysis to infer whether or how it could impact the last mile, particularly in absence of downstream analysis of the specific program and situation. Thus, while additional research is needed to examine review delays occurring in the last mile, alleviating first mile issues will be a step in the right direction to improve MDA timeliness. Additionally, this review of improvements to first mile performance aligns with the focus of the WHO to improve on-time delivery to central medical stores.

However, we acknowledge the importance of the last mile to the NTD supply chain. Due to the limited information and data about the last mile, we discuss this as a limitation and further research direction. A statement was added in the methods section to more explicitly provide this rationale for the value of improving first mile performance: *“While the last mile certainly involves many challenges that contribute to MDA delays, issues with the first mile have shown in practice and research to have a downstream effect on the last mile and ultimately MDA timeliness - thus, alleviating first mile issues will be a step in the right direction to improve MDA timeliness.”* Additionally, a statement was added in the discussion of limitations to reflect the consideration of additional focus needed on last mile improvements: *“Also, incorporating any data pertaining to the last mile aspect of the supply chain in this tool might help to improve those downstream processes, which remains a priority, given the last mile’s impact on efficient and effective distribution and planning for MDAs.”*

***“In addition, it does not seem logical to evaluate timeliness as a continuous measure – it probably makes no difference if the drugs are in the central warehouse 6 months or 5 months ahead of MDA – what matters is whether they are there and can be distributed in time for MDA. I would think a binary cutoff based on what this sufficient time is would be more practical.”***

Thank you for this consideration as this was in fact a decision carefully reviewed and considered when setting up the research design. This decision to keep the variables as continuous measures, rather than changing to binary measures, was made based on both statistical and practice considerations. In terms of statistical considerations, it is generally not recommended to convert continuous variables into binary variables unless there are compelling reasons [2,3,4]. Particularly, “dichotomising” variables may significantly reduce statistical power and could lead underestimating the level of variation in outcome between groups [2,3,4,5]. In this case, a shipment delivered 28 days prior to MDA effectively would be in the same group as a shipment delivered months after the MDA date.

More importantly, from a practice perspective, there is in fact value for the shipment to arrive ahead of the MDA program, as this allows greater flexibility in planning downstream distribution and logistics, especially in larger countries with complex health infrastructure. This consideration in part explains why the WHO recommends a standard for delivery to central medical stores of two months before the planned MDA date yet accepts a minimum standard of one month: the one month is considered feasible for last mile delivery but the two months is more optimal as it affords more time for such downstream planning [6]. Therefore, there is arguably more value to review these metrics as continuous measures since the performance measure is not truly a binary assessment that results in classifying as either “on-time” or “late.”

***“Finally, some of the “improvements” may be statistically significant but that does not make them programmatically relevant. I am not sure a two week improvement is meaningful for example.”***

In the writing of the manuscript, we aimed to keep the statistical analysis and discussion of implications for practice separate. The results appear to suggest a significant improvement anywhere from about a month to over two months depending on the variable, as opposed to two weeks. These results would likely have implications for practice as well; as noted, there is value for the shipment to arrive sooner ahead of the MDA for greater flexibility in planning distribution. Though incremental, the process appears to be headed in the favorable direction. Thus, there may be potential for further improvements in timeliness. Aside from the observed improvement, there is also the implication for practice that information sharing appears to positively impact performance measures. Such a finding could possibly generate further programmatically relevant implications for NTD programs beyond a supply chain scope, although certainly further research would be needed to confirm a broader application.

***“The paper is written from the standpoint of a data analyst and much of the language is difficult to follow and not clear to the intended audience. The way in which the analysis is presented is not intuitive and does not align with metrics that the audience will want to understand.”***

Thank you for this suggestion. We have made efforts to revise the language throughout the paper, as detailed in our detailed responses to other comments. The intent was to reserve the analytical language to the results section and focus on interpretation of the data relevant to our hypotheses within the discussion section. We hope the current version is easier to read and follow for boarder audience.

***“The authors seem to erroneously attribute magnitude of significance with the p value in their analysis. First, it is not at all clear why a non-standard p value of .1 was chosen to represent significance, particularly given that the study design is already weak. This seems likely to have been a post-hoc decision made following a look at the results, particularly given that many analyses are right at the 0.9 level. Please revise to use a standard p value of 0.5 for significance. In addition, something is not “more significant” if its p value is smaller. The key metric is the effect size, and then whether or not it meets the threshold for significance. Please revise the text accordingly.”***

Thank you for the suggested edits. The threshold for significance was changed from 0.10 to 0.05, as suggested. Due to this edit, arrival timeliness in the main results addressing the first research question is no longer considered significant against this new threshold and the text was updated accordingly. Otherwise, all other results met the p <0.05 threshold for significance.

In terms of the note on revising the language implying a smaller p-value indicates a “more significant “association, note the following changes made to reflect this feedback:

- Revisions were made to the language in the abstract and introduction, such as changing the wording from *“there is an increased positive impact when the data is publicly shared” to “there is a positive association with improvement for three variables when the data is publicly shared”*
- Many revisions were made to the language and results and discussion section, such as changing *“shipment timeliness is the most significant variables at a p-value of 0.001” to “shipment timeliness is significant at a p-value of 0.001”*

***“The results and the discussion need to be separated. There is far too much discussion included in the results section. Please just present the results cleanly and then interpret them in the discussion.”***

Thank you for the feedback – to address this point, significant revisions were made to move statements related to interpretation of the results from the results to the discussion section.

***“The weaknesses of this study design are a major limitation (as noted by the authors). There is no reason why an RCT or a stepped wedge study could not have been designed in coordination with WHO. One key takeaway in the conclusion is the need to plan robust evaluations from the start so that more meaningful impact assessment can be conducted.”***

This study was designed and initiated a few years after the system had been implemented and therefore randomization was a significant challenge. As the system was not rolled out in a phased approach and was rolled out for all countries and donations simultaneously, identifying a true “control group” – randomly selected or otherwise – was also a challenge. A quasi-experiment design, using a “one-group pretest-posttest design without control group,” was used to leverage historical data available on performance to understand the impact of this intervention. Also, although there is no “control group” in our research design, we conduct a “double pretest” to investigate if other observed time-varying variables may contribute to the significant results. We revised the methods section to clarify that the research was initiated a few years after the launch of the tool and this in part contributed to the limitation of the ability to randomize. As well, in the discussion section, it is noted that future research could study the impact of information sharing in a controlled setting with randomization incorporated in the design. In that case, the findings reported here should support and guide the development of a controlled study in the future.

***“I was confused by the language around confounders and controls – these are*** ***important cofactors or mediators of effect – not confounders.”***

Thank you for this comment. Note that the text under the methods section referencing these variables has been updated to incorporate this feedback via the following updated sentence: *“Important cofactors, incorporated via control variables, were included in the analyses to explore whether the relationship of the independent and dependent variables is skewed or invalidated by other factors.*”

***“******The data could be presented graphically or at least in some much more readily digestible format. The raw output of the regression analyses are not the best way to present these data to the intended audience.”***

Thank you for this feedback. We have made some modifications to the format of the results tables to provide a more simplified view and better align with the format of regression results recently published by other authors in *PLOS NTDs*. The papers used for referenced to make these formatting adjustments include Mandro et al.’s 2020 study, Amare and Lindtjørn’s 2020 study, Costa et al.’s 2021 study, and Brintz et al.’s 2020 study [7,8,9,10]. To highlight the nature of the updates made to the presentation of the results, see below the revised format of Table 2 providing the regression results showing the relationship between NTD supply chain key performance indicators and implementation of NTDeliver.

| Variables | N | Β reg coefficient | p-value | Predicted direction | Actual direction |
| --- | --- | --- | --- | --- | --- |
| PO timeliness  (in months) | 897 | 0.94 | 0.003 | ↑ | ↑ |
| Go signal timeliness  (in days) | 216 | 41.483 | <.001 | ↑ | ↓ |
| Shipment timeliness  (in months) | 535 | 0.033 | 0.927 | ↑ | ↑ NS |
| Arrival timeliness  (in months) | 661 | 0.516 | 0.129 | ↑ | ↑ NS |
| Delivery timeliness  (in months) | 528 | 0.828 | 0.027 | ↑ | ↑ |
| Abbreviations: Reg, regression; CI, confidence interval; NS, non-significant  Incorporated control variables: WHO region, international LPI, medicine type, disease, order size, mode of shipment | | | | | |
| Regression results showing the relationship between NTD supply chain key performance indicators and implementation of information sharing through NTDeliver | | | | | |

***“Small issue – data are pleural – datum is singular – update text accordingly.”***

Thank you for this note. The text has been revised to reflect where the usage of the term “data” is intended as the plural by editing the text in numerous areas from “data is” to “data are” and similarly editing “data was” to “data were.”

**III: Detailed Response to Reviewer 2**

***“Were there any time-varying factors besides the NTDDeliver and NTDPublic which may be coincident with the study time frame. Countries, manufacturers, and WHO are constantly trying new improvements. Not clear how the study design robustly controls for that?”***

Many thanks for this valuable comment. Particularly as the NTD Supply Chain Forum has limited visibility or specific documentation of the country-level logistics since the focus is on the first mile, gaining visibility and controlling for all initiatives at the country-level is not possible. A country-based analysis would likely entail a project with a much larger order of magnitude, requiring resources and access to data and country-specific variables. However, because the data encompasses over 100 countries, even if a country pursued specific improvement initiatives, we would expect the impact to the results to be insignificant since the data sample is very diversified. This is also in part why we believe controlling for region is more impactful: regional-level improvements are more likely to impact the results since such efforts impact a great scope of the countries, and therefore a great scope of the data sample. In addition, the WHO is subdivided into regional offices and thus may impart management decisions that have impact on the region as a whole.

Additionally, we believe the robustness checks conducted (discussed briefly in the manuscript, with results included in the appendix in Table A4 also provide more confidence in the intervention of the NTDeliver system being attributed to the observed results, as opposed to other initiatives. The robustness check of considering a six-month lag supports that even information sharing effect took time to make an impact, the performance still improved and can be attributed more confidently with the implementation of NTDeliver. Additionally, the comparison of performance before the information sharing through NTDeliver was implemented (i.e. the “double pretest”) suggests that the supply chain performance does not simply improve over time and did not reveal any significant changes to performance (other than PO timeliness, which we believe is likely explained by the implementation of the “joint application package,” as discussed in the manuscript), even though there were also supply chain improvement initiatives in the timespan before NTDeliver was implemented.

However, per the additional feedback provided in a later comment to account for country logistics factors, we did incorporate the Logistics Performance Index (LPI) benchmark tool that the World Bank assesses to account for any country-level logistics factors. Therefore, incorporating this LPI score as an additional control variable may also help control for any improvements that occurred at a country level. The details of the incorporation of this variable are addressed in the answer to the subsequent question regarding country logistics factors.

***“Presumably there is a higher likelihood of missing data in the pre-intervention sample. So without explicitly showing that missing data would be completely random (and covariance matrix etc.) it is unclear how the study design removes biases that may result from non random missing data.”***

Thank you for inquiring on this important consideration regarding missing data. As part of the data preparation, a review on missing data was conducted to better understand how the data availability accounted for differences in the representation of the dependent variables between the “pre” and “post” sets. Missing data in the system is indicated by “N/A,” but this does not exclusively represent missing data and sometimes indicates that the shipment is too early in the process for data availability. This was more applicable to the “post” data, since some shipments’ data set was not yet completed if delivery had not progressed much further than the PO. For at least the two dependent variables with clear performance benchmarks, if the dependent variables had an “N/A” fitting the criteria below and had an “N/A” for all downstream KPIs, this was not considered indicative of truly missing data:

- Shipment date: “PlannedPC” later February 2019 for air/land shipments or later than April 2019 for sea shipments, since the one- or three-month pre-MDA benchmark has not yet been missed
- Delivery warehouse: “PlannedPC” later than February 2019, since the one-month pre-MDA benchmark has not yet been missed

While PO timeliness has a clear benchmark, all shipments had a “PO raising date”, unless the order was cancelled, which could be confirmed in the system by a total order value of “0”. The data was cleaned in the “post” group for the “N/As” and “0s” fitting these criteria to create visual bar charts to comparing missing data for each dependent variable. The review distinguishes between “pre” and “post” data differences, and then reviews for differences in data attributes (region, medicine, disease). The graph below provides a visual view of the percent of data availability for each dependent variable, comparing the “pre” and “post” data sets.

% of Available Data, by "Pre" and "Post" SCPMS

|  | Post-SCPMS | Pre-SCPMS |
| --- | --- | --- |
| Planned PC | 95% | 64% |
| Delivery warehouse date | 65% | 44% |
| Actual arrival | 66% | 64% |
| Shipment date | 74% | 32% |
| Go signal approved | 32% | 11% |
| Go signal request | 34% | 12% |
| PO raised | 100% | 100% |

***Figure: Percent of available data for each dependent variable***

Calculations for the percentages were conducted using the respective totals from the “pre” and “post” data sets – i.e., the % of “PO raised” data in the “pre” data set was calculated by taking the total amount of shipments with this data point available out of the denominator of total data in the “pre” data set. Variables such as “PO raised” and “actual arrival” have a very close match in data percentage availability between the “pre” and “post” data sets. However, “PO raised” is available for a greater percentage, at nearly 100% availability for both data sets, while “actual arrival” is only available for a little over 60%. Also, differences are evident in availability between the “pre” and “post” data sets. “Shipment date” has one of the most dramatic contrasts, with the “pre” data set only having this data point available for a little over 30% of shipments, and the “post” set over 70% of shipments. Delivery warehouse date, one of the most critical pieces of data for the research, also has a difference in availability between the data sets, although less dramatic than shipment date. Additionally, an analysis was conducted to determine if there was a trend in the missing data from the variables that may indicate the data is non-randomly missing. To understand whether variables were related to missing values from another variable, a new variable indicating whether a value was missing (0,1) was created and used to compare performance for any significance in difference. From review, no systematic or clear pattern was observed, supporting that missing data is missing as random and will not significantly contribute to trend in the model [11]. Since data was found to be missing at random and there is a significant sample size even for the dependent variables with the smallest samples, imputation did not need to be applied and pairwise deletion of the missing data was a sufficient approach.

The covariance matrix is also provided below:

|  | | PO Timeliness (in months) | Go Signal Timeliness  (in days) | Shipment Timeliness  (in months) | Arrival Timeliness  (in months) | Delivery Timeliness  (in months) |
| --- | --- | --- | --- | --- | --- | --- |
| PO Timeliness (in months) | Pearson Correlation | 1 | .205^**^ | .659^**^ | .704^**^ | .675^**^ |
|  | Sig. (2-tailed) |  | .003 | .000 | .000 | .000 |
|  | N | 1070 | 212 | 635 | 792 | 639 |
| Go Signal Timeliness (in days) | Pearson Correlation | .205^**^ | 1 | .126 | .131 | .142 |
|  | Sig. (2-tailed) | .003 |  | .073 | .067 | .056 |
|  | N | 212 | 246 | 202 | 196 | 183 |
| Shipment Timeliness (in months) | Pearson Correlation | .659^**^ | .126 | 1 | .963^**^ | .927^**^ |
|  | Sig. (2-tailed) | .000 | .073 |  | .000 | .000 |
|  | N | 635 | 202 | 635 | 571 | 543 |
| Arrival Timeliness (in months) | Pearson Correlation | .704^**^ | .131 | .963^**^ | 1 | .939^**^ |
|  | Sig. (2-tailed) | .000 | .067 | .000 |  | .000 |
|  | N | 792 | 196 | 571 | 792 | 574 |
|  | Sig. (2-tailed) | .039 | .895 | .074 | .012 | .003 |
|  | N | 212 | 103 | 209 | 211 | 210 |
| Delivery Timeliness (in months) | Pearson Correlation | .675^**^ | .142 | .927^**^ | .939^**^ | 1 |
|  | Sig. (2-tailed) | .000 | .056 | .000 | .000 |  |
|  | N | 639 | 183 | 543 | 574 | 639 |
|  | Sig. (2-tailed) | .019 | .869 | .008 | .467 | .001 |
|  | N | 353 | 173 | 343 | 352 | 345 |

**Correlation is significant at the 0.05 level (2-tailed)*

***Correlation is significant at the 0.01 level (2-tailed)*

***“An important control variable which is missing is country characteristics e.g. country logistics factors, etc. What if there was a differences in the countries receiving the orders in the pre-and post intervention phase. The authors have controlled for WHO region, but that is too coarse a variable to account for country specific differences”***

Thank you for the valuable comments. Overall, in terms of the differences of the countries receiving orders in the “pre” and “post” set, the differences in representation at both the regional and country-level are very marginal. The table below compares the representation of regions in each data set. As seen, the differences are minimal, with only a small percentage of variation. Some variation between the data sets would be expected as more countries in select regions achieved their targets, such as in the case of WPRO.

| Region | % of “Pre” Data | % of “Post” Data |
| --- | --- | --- |
| AFRO | 47.0% | 47.6% |
| AMRO | 6.2% | 6.3% |
| EMRO | 4.0% | 7.5% |
| EURO | 2.6% | 1.4% |
| SEARO | 24.5% | 27.6% |
| WPRO | 15.4% | 9.1% |
| N/A | 0.3% | 0.5% |
| TOTAL | 100.0% | 100.0% |

A comparison of representation at the country level was also conducted. This review showed each given country found in both data sets tends to have roughly the same representation percentage: there is no more than 1% difference in representation for any given country. The exception to this is only for three countries where there is greater than a 1% difference in representation: 1) Cameroon represents 1.4% less in the “post” set compared to the “pre” set; 2) Egypt represents 1.4% more in the “post” set than the “pre” set; and 3) Somalia represents 1% more make-up in the post set compared to the “pre” set. Lastly, countries in the “post” set but not in “pre” set make up less than 2% of total “post” data and account for 3 countries total. Countries in the “pre” set not in “post” set make up ~4% of total “pre” data and account for 11 countries total.

With regard to the consideration of controlling for country logistics factors, as previously mentioned the NTD Supply Chain Forum has limited specific documentation or significant visibility of the country-level logistics since the focus is on the first mile. Also as mentioned, we do believe controlling for region is in some sense more impactful since regional-level improvements are more likely to impact the results since such efforts impact a great scope of the countries, and therefore a great scope of the data sample. However, we can incorporate an external assessment of country logistics factors as a control variable. We believe the international Logistics Performance Index (LPI) benchmark tool that the World Bank assesses is a relevant indicator to help control for such factors. The LPI measures performance of the logistics supply chain within a country, with the international LPI providing a qualitative evaluation by trading partners of the country’s logistics performance [12]. The international LPI is more relevant to the scope of the first mile than the domestic LPI measured, as the first mile is more oriented with import into the country, as opposed to internal country logistics.

To incorporate the LPI, the overall LPI score for a given country was included in the data. The LPI is not assessed every year, therefore the score for the year of assessment closest to the year of the purchase order was included and assigned according to the table below.

| Purchase Order Year | LPI Year |
| --- | --- |
| 2006 | 2007 |
| 2007 | 2007 |
| 2008 | 2007 |
| 2009 | 2010 |
| 2010 | 2010 |
| 2011 | 2012 |
| 2012 | 2012 |
| 2013 | 2014 |
| 2014 | 2014 |
| 2015 | 2016 |
| 2016 | 2016 |
| 2017 | 2018 |
| 2018 | 2018 |

Additionally, the LPI score is not available for all countries, but an available score was incorporated for 1268 0f 1484 shipments (85%)– therefore, the majority of shipments had this variable included. The regression analysis was rerun with this new control variable included. Overall, the results did not substantially change. There were some slight changes in the coefficient and p-values, but the KPIs that were significant in the original analysis remained significant. The number of observations did decline a bit, given that the LPI score was not available for all countries.

| Dependent Variable | PO timeliness  (in months) | Go signal timeliness  (in days) | Shipment timeliness  (in months) | Arrival timeliness  (in months) | Delivery timeliness  (in months) |
| --- | --- | --- | --- | --- | --- |
| NTDeliver | 0.94 | 41.48 | 0.03 | 0.52 | 0.83 |
| p-value | (0.003) | (0.001) | (0.93) | (0.13) | (0.03) |
| Predicted direction | ↑ | ↓ | ↑ | ↑ | ↑ |
| Actual direction | ↑ | ↑ | ↑ NS | ↑NS | ↑ |
| WHO region | Yes | Yes | Yes | Yes | Yes |
| International LPI | Yes | Yes | Yes | Yes | Yes |
| Medicine type | Yes | Yes | Yes | Yes | Yes |
| Disease | Yes | Yes | Yes | Yes | Yes |
| Order size | Yes | Yes | Yes | Yes | Yes |
| Mode of shipment | Yes | No* | Yes | Yes | Yes |
| Observations | 897 | 216 | 535 | 661 | 528 |
| NS = non-significant  *** Sample size too limited to incorporate variable | | | | | |
| Regression results showing the relationship between NTD supply chain key performance indicators and implementation of information sharing through NTDeliver | | | | | |

***“Did the authors explore the use of a RD type study design?”***

Thank you for this good question. Because our study was designed and initiated a few years after the system had already been implemented, randomization was not possible. Since the system was not rolled out in a phased approach and was implemented for all countries and donations, a true “control group” – randomly selected or otherwise – was also a limitation. A quasi-experiment design, using a “one-group pretest-posttest design without control group,” was used to leverage historical data available on performance to understand the impact of this intervention. Also, although there is no “control group” in our research design, we conduct a “double pretest” to investigate if other observed time-varying variables may contribute to the significant results. We have revised the methods section to clarify that the research was initiated a few years after the launch of the tool and that this in part contributed to the limitation of the ability to randomize.

***“The result are difficult to interpret without a clear exposition of what each of the time variables means ie. when does the clock start for each timeliness variables and then does the clock stop. The authors should consider creating a diagram with the timelines and variable definition on that.”***

Thank you for this comment. Figure 2 has been added to the manuscript to provide further clarity on the variables with benchmarks (i.e., a targeted “start/stop” on the clock) and which are in scope/out of scope of the study. A copy of this figure added to the manuscript is provided below for reference.

Application

At least 8 months prior to MDA

Purchase Order

At least 6 months prior to MDA

Go Signal

Shipment

At least 3 months prior to MDA

Arrival

Delivery (to Central Medical Store)

At least 1 month prior to MDA

Last Mile Delivery to MDA

**Fig 2.** **Simplified timeline of the NTD supply chain, associated benchmarks, and scope of research**

Components in scope of first mile and research review

**IV: Detailed Response to Reviewer 3**

***“Based on the findings can the authors further clarify which stakeholder behavior changed based on the variable. E.g., Did PO timeliness reflect WHO management? Did shipping times reflect the pharmaceutical companies. Were these results possibly influenced by other stakeholders indirectly such as NGOs supporting governments efforts?”***

Thank you for this comment. Because our research was purely a quantitative design to understand if supply chain performance significantly changed as a result of introducing the system and did not focus on assessing whether any observed performance changes were due to changes in stakeholder behaviour, it would be challenging to extrapolate from the data if behaviour change was a direct cause for the observed results. While the increased visibility to the data could have very well changed how stakeholders performed in their role in the supply chain knowing the visibility increased (i.e. the WHO raising purchase orders more quickly or pharmaceutical companies shipping donations sooner than historical performance, knowing the data is now publicly viewable), it is also possible that the increased visibility to the data increased the opportunity for process improvements (i.e. being able to see real-time updates on POs and prioritizing more effectively). This question of stakeholder behaviour changes as a result of information sharing/visibility would be an interesting question to explore outside the scope of this research with qualitative research using interviews and/or surveys to investigate stakeholder behaviour changes. A sentence was added to the manuscript under the discussion section to acknowledge this possible future research area: *“Also, modifications to the design could also entail incorporating qualitative research using interviews and/or surveys to investigate whether changes in stakeholder behaviour are associated with the observed performances changes.”*

***Editorial suggestion provided for the following statement in the text: “While significant progress was made towards these 2020 targets, the WHO has recently released a new NTD roadmap with 2030 targets, which pharmaceutical manufacturers have committed to continuing to support [2].” Suggestion provided on this statement: “Pharmaceutical companies support the road map but not all donation programs have committed to donating pharmaceuticals through 2030”***

Thank you for this clarification. We have edited the statement accordingly to read that “many” pharmaceutical manufacturers have committed to continuing to support.

***Editorial suggestion provided for the following statement in the text: “Delivery delays result in waste, increased program costs, and delayed or even completely missed MDAs, leaving individuals susceptible to NTD infections [7].” Suggestion provided on this statement: “More accurate to state ‘…..leaving communities susceptible to disease recrudescence.’”***

Thank you for this suggestion to clarify the impact of delivery delays – the suggested update has been made to the text. The phrase “infections” was also added since missed MDAs leave communities susceptible to both "infections and disease recrudescence."

***Editorial suggestions provided on the following figure: “Figure 1. Components of the NTD supply chain from application for donation to in-country delivery to central medical stores”. Suggestion provided on this statement:***

***-       Application: performance measure is 8 months prior to MDA (not 6)
-       Manufacturing:*** ***performance measures are variable by manufacturer
-       Shipment: three months prior to MDA is the UNICEF standard of 10 years ago for sea freight. This is not WHO policy.
-       In-country processes: Performance measures should say varies by country***

The six months in the figure intended to refer to the standard for PO issue, rather than the application, but perhaps may not have been very clearly expressed. To alleviate any ambiguity, the text was updated to the following: *“Process: application/order finalized at country/regional level (8 months prior to Mass Drug Administration - MDA); WHO HQ raises purchase order to the manufacturer (6 months prior to MDA).”* The suggested edit was made to the manufacturing process description. In regard to the shipment process, the three-month shipment standard is followed within the NTD Supply Chain Forum, despite that is not an officially documented WHO policy publicly. The memorandum of understanding (“MOU”) agreement that each pharmaceutical company has with the WHO does document this three-month standard for shipments, but because this is a proprietary agreement that is not public, we cannot reference this in the manuscript. Lastly, the following statement was added to update the in-country processes performance details: *“other last mile performance measures may vary by country.”*

***“Clarify the NTD Supply Chain Forum membership. If stating the present Forum makeup, some members are missing. If wishing to indicate the makeup at the time of establishment, please make this clear.”***

Thank you for this comment. We have edited the introduction to the NTD Supply Chain Forum membership to a broader description of membership to reduce the risk of omission of any specific members. The source for the updated statement comes from a very recent viewpoint article published in PLOS Neglected Tropical Diseases by members of the Forum itself [13]. The revised statement in the manuscript is the following: *“The NTD Supply Chain Forum is comprised of NTD supply chain experts from the WHO, pharmaceutical companies, nongovernmental organizations, donor organizations, ministries of health, and logistics providers.”*

**References**

1. Koporc KM, Strunz E, Holloway C, Addiss DG, Lin W. Assessing “first mile” supply chain factors affecting timeliness of school-based deworming interventions: Supply and logistics performance indicators. *PLOS Neglected Tropical Diseases.* 2015;9(12).
2. Altman DG, Royston P. The cost of dichotomising continuous variables. *BMJ.* 2006;332(7549):1080.
3. Royston P, Altman DG, Sauerbrei W. Dichotomizing continuous predictors in multiple regression: a bad idea. *Stat Med*. 2006 Jan 15;25(1):127-41.
4. MacCallum R, Zhang S, Preacher K, Rucker D. On the practice of dichotomization of quantitative variables. *Psychological Methods.* 2002;7(1):19-40.
5. Lamb A, Smilack J, Ho A, and Reich J. 2015. Addressing Common Analytic Challenges to Randomized Experiments in MOOCs: Attrition and Zero-Inflation. In Proceedings of the Second (2015) ACM Conference on Learning @ Scale (L@S '15). Association for Computing Machinery, New York, NY, USA, 21–30.
6. Weekly Epidemiological Record, 31 March 2017, vol. 92, 13 (pp. 145–164). World Health Organization. World Health Organization; 2017. Available from: https://www.who.int/wer/2017/wer9213/en/
7. Mandro M, Siewe Fodjo J, Mukendi D, Dusabimana A, Menon S, Haesendonckx S et al. Ivermectin as an adjuvant to anti-epileptic treatment in persons with onchocerciasis-associated epilepsy: A randomized proof-of-concept clinical trial. *PLOS Neglected Tropical Diseases.* 2020;14(1):e0007966.
8. Hailu Amare H, Lindtjørn B. Helminth infections among rural schoolchildren in Southern Ethiopia: A cross-sectional multilevel and zero-inflated regression model. *PLOS Neglected Tropical Diseases.* 2020;14(12):e0008002.
9. Costa F, Zeppelini C, Ribeiro G, Santos N, Reis R, Martins R et al. Household rat infestation in urban slum populations: Development and validation of a predictive score for leptospirosis. *PLOS Neglected Tropical Diseases.* 2021;15(3):e0009154.
10. Brintz B, Howard J, Haaland B, Platts-Mills J, Greene T, Levine A et al. Clinical predictors for etiology of acute diarrhea in children in resource-limited settings. *PLOS Neglected Tropical Diseases*. 2020;14(10):e0008677.
11. Salkind, N. J. (2010). *Encyclopedia of research design* (Vols. 1-0). Thousand Oaks, CA: SAGE Publications, Inc. doi: 10.4135/9781412961288
12. Logistics Performance Index [Internet]. Home | Logistics Performance Index. The World Bank; [cited 2021Oct26]. Available from: https://lpi.worldbank.org/
13. Souza AA, Holloway C, Williams T. The NTD Supply Chain Forum—Strengthening the backbone of NTD programs. *PLOS Neglected Tropical Diseases.* 2020;14(11).
